# Study on failure mechanism of tight sandstone based on moment tensor inversion

**DOI:** 10.1016/j.heliyon.2023.e19030

**Published:** 2023-08-16

**Authors:** Yike Dang, Zheng Yang, Haiyan Zhu

**Affiliations:** a School of Human Settlements and Civil Engineering, Xi'an Jiaotong University, Xi'an, 710049, China; b State Key Laboratory of Oil and Gas Reservoir Geology and Exploitation, Chengdu University of Technology, Chengdu, 610059, China

**Keywords:** Acoustic emission, Moment tensor inversion, Failure evolution, AE magnitudes, Tight sandstone

## Abstract

Understanding deep rocks' mechanical properties and failure evolution is crucial for efficient resource development. This study investigates the mechanical properties of tight sandstone and analyzes its acoustic emission (AE) characteristics using a combined discrete element model and moment tensor inversion. The AE activity during loading is categorized into three stages: crack initiation, stable crack propagation, and unstable crack propagation. Confining pressure loading suppresses AE activity during the crack initiation stage due to damage healing phenomenon. Moment tensor inversion reveals that tensile failure is the primary AE failure source, despite samples exhibiting splitting and shear failure modes. The proportion of AE failure types varies with stress levels and depends on the mechanical environment. Microcracks initiate at the ends of the sample and propagate inward along the loading direction, resulting in a blank area of AE events in the middle. This blank area can be utilized to predict specimen failure mode. The *b* value, representing the ratio of small to large magnitude events, decreases with increase of the confining pressure, indicating higher energy release during specimen failure under high confining pressure. The research results can provide a reference for predicting the failure of tight sandstone.

## 1 Introduction

With the increasing depletion of shallow resources, the development of coal, oil and other mineral resources has gradually entered the stage of deep mining. The disturbance caused by underground construction will cause the growth and expansion of cracks in the surrounding rock. It will reduce the structural integrity and bearing capacity of a rock. Due to the research of deep rock mechanics is far behind the practice of deep engineering. Therefore, it brings great trouble to the construction [1], [2], [3]. The generation of fractures in the rock mass is the early stage of various disasters during excavation. Monitoring the propagation of rock fractures is of great significance for deeply understanding the failure mechanism of rock, efficient development of natural resources, and disaster prevention and reduction.

At present, a large amount of research has been conducted on the mechanical properties of sandstone. Liang et al. [4] studied the effect of water content on the mechanical behavior of sandstone under triaxial compression. The results indicate that long-term saturation increases the strain softening of sandstone, thereby increasing its deformation and reducing its strength. Huang et al. [5] investigated sandstone's mechanical properties and failure characteristics under different confining pressures. Research has shown a positive correlation between peak strength and confining pressure. The increase in confining pressure promotes the densification of internal defects, such as pores and cracks in the rock sample, which increases the stiffness of the rock. Therefore, the elastic modulus of the rock increases accordingly. The failure mode of the sample gradually transitions from tensile failure to shear failure with the increase of confining pressure. You et al. [6] conducted triaxial dynamic tests on sandstone containing defects and studied the effects of confining pressure and strain rate on its mechanical properties. The results indicate that the dynamic elastic modulus of sandstone exhibits a significant confining pressure enhancement effect while not sensitive to the corresponding strain rate. The dynamic strength is positively correlated with confining pressure and strain rate. Zhao et al. [7] investigated the mechanical properties of sandstone under confining pressure cyclic loading and unloading conditions. The experimental results show that the elastic modulus and Poisson's ratio of sandstone increase significantly with the rise of initial confining pressure and decrease in gradient during the whole cyclic loading and unloading process. Under high confining pressure, the stress state of the sample is improved, which hinders the further propagation of microcracks. Huang et al. [8] studied the fracture toughness of sandstone under confining pressure. The sensitivity of failure load, fracture toughness, and crack growth to confining pressure and geometric parameters of the specimen were analyzed. The results show that the Fracture toughness of sandstone is linearly related to the confining pressure. Liu et al. [9] studied the mechanical properties of rocks containing defects under triaxial tension. The results indicate that confining pressure significantly impacts the failure strength of rock-containing defects. The failure strength and axial deformation of the sample increase with the confining pressure, and the failure stress gradually changes from tensile stress to compressive stress.

There are more or less primary defects in solid materials such as rock. External forces will activate the internal defects, causing damage and destruction. In the process of damage and destruction, strain energy will be released in the form of elastic waves and spread rapidly inside the rock mass, resulting in Acoustic Emission (AE) [10]. Rock AE is now an accompanying phenomenon of rock failure. It contains much information on the failure process of the rock. Study the relationship between AE parameters and mechanical properties during rock failure. Understanding the rock failure mechanism and providing a practical basis for further prediction of rock failure and instability is helpful [11], [12], [13], [14], [15].

Many scholars have studied the AE characteristics in the process of rock failure. Kong et al. [16] based on the AE methods to study the damage evolution and mechanical properties of coal, the results can help understand the effects of methane on coal, and it can be applied to research on the early warning of gas outburst. To investigate the AE characteristics of shale gas rocks. Luo et al. [17] studied the dynamic failure of rock through true triaxial test combined with AE technology. The results show that the rock failure is related to the dynamic disturbance of fatigue damage effect and elastic strain energy release. Li et al. [2] adopted the AE to reveal AE's spatial distribution and hypocenter mechanisms. Wang et al. [18] conducted laboratory tests of water-saturated tuff under triaxial cyclic compression and discussed the fatigue behavior of water-saturated tuff. Wang et al. [11] investigated rock-like materials' mechanical and AE characteristics under non-uniform loads. They found that rock-like materials' mechanical and AE characteristics can be notably localized. Zhai et al. [19] analyzed the fracture evolution during rock bursts of granodiorite and basalt based on the AE methods. Zhang et al. [20] carried out the AE system to analyses the confining pressure and cleat orientation influence on the coal brittleness. Zhang et al. [21] conducted the true triaxial loading test to study gas-bearing coal's deformation and failure characteristics by the AE system. The research result boasts instructive significance for preventing the occurrence of coal. Zhou et al. [22] used the AE system to estimate the rock damage evolution and proposed the simplified models of failure process for crystalline rocks under compression and HM coupling. Hampton et al. [23] used AE technology to monitor the initiation and expansion process of hydraulic fractures during hydraulic fracturing tests and adopted separate AE events and sources to characterize the failure mechanism of samples. Dong et al. [24] explored the influence of intermediate principal stress on rock failure process and the precursor characteristics of rock instability by analyzing the characteristic parameters of AE, and analyzed the identification method of the direction of rock principal stress under complex stress states. They conducted research on the formation and propagation mechanism of microcracks in the evolution of granite failures, the research results provided insights for predicting the precursors of rock instability [25]. Ding et al. [26] studied the AE behavior of pre-cracked coal under different loading conditions through graded loading experiments. The relationship between AE events and coal damage instability was explored, and its mechanism was explained. Wen et al. [27] proposed a new method for locating AE sources, which combined with moment tensor inversion results to elucidate the failure mechanism of grouting specimens containing random inclusions. Zhao et al. [28] investigated the failure characteristics of rocks containing defects. The results indicate that the number, energy, and distribution of AE events are closely related to the failure mode and instability process of rocks.

The AE technology has often encountered some problems, such as the acoustic sensor is not sensitive, and it is easily damaged by impact, resulting in incomplete or impossible data recording, which also limits the application of this method. As an auxiliary research method, numerical simulation has been widely used by more and more scholars because it can set up complex experimental conditions to supplement indoor experiments. Tang et al. [29] proposed a numerical approach that can be modeling progressive failure of rock, the program can calculate the evolution of damage and AE events due to progressive failure in brittle rock. Lisjak et al. [30] describes a new AE modeling technique, it can consider fracture nucleation and propagation in the rock failure. The Discrete element method (DEM) is a numerical simulating method based on a discontinuous medium. It has unique advantages for subjects such as fracture propagation and mesoscopic problems. Hazzard and Young [31], [32] introduced a method for simulating AE in the Particle Flow Code (PFC), there were obtained the magnitude of AE by a reasonable calculation method. Hunt et al. [33] conducted the PFC model to reproduce the Kaiser effect, and the deformation rate analysis phenomenon was confirmed. Chong et al. [34] based on the moment tensor to study the AE characteristics of transversely isotropic by DEM-AE model. Xu et al. [35] used a numerical model to distinguish the crack nature in the bridge region of two pre-existing flaws. The DEM-AE model are used to study the AE characteristics and failure mechanism of rock containing hole-like flaw by Zhang et al. [36]. Zhang et al. [37] analyzed the failure process of brittle rock based on the moment tensor. Samin et al. [38] studied seismic source mechanisms of AE events generated during the failure of intact and jointed rock samples using a microscale mechanical-seismicity coupled microscale model.

Scholars at home and abroad have conducted extensive research on deep rock materials' AE characteristics and achieved some results. However, there are some shortcomings in indoor AE research [39]. The probe is not sensitive enough, and the AE signal is difficult to capture under high confining pressure. It limits our understanding of the AE characteristics of deep rocks. In order to better understand the fracture process and AE characteristics of rock under high confining pressure, firstly, the uniaxial and triaxial compression of tight sandstone is carried out in this paper. Then, combined with the discrete element model and moment tensor inversion, the AE characteristics and failure process under high confining pressure are quantitatively analyzed. This study provides an idea for studying the formation process of deep underground fractures such as sudden failure of the tunnel, rock burst and wellbore instability.

## 2 Experimental materials, methods and results

### 2.1 Experimental system

**Figure 1:**
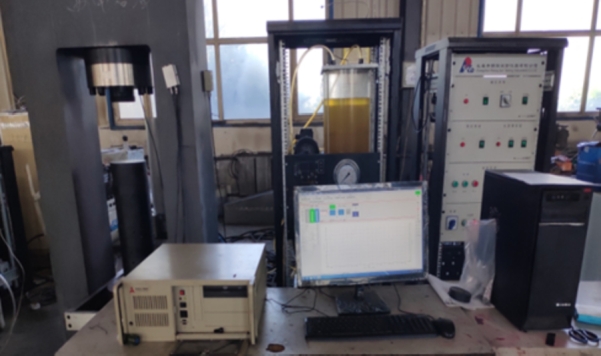
TAW-2000 multi-field coupling experimental system.

The experiment system adopts the TAW-2000 multi-field coupling test produced by the Changchun Chaoyang test instrument company. The maximum axial pressure of the equipment is 2000 kN. The hydraulic servo applies the confining pressure. The maximum value can be applied to 100 MPa. The experimental system is shown in Fig. 1.

### 2.2 Sample preparation

**Figure 2:**
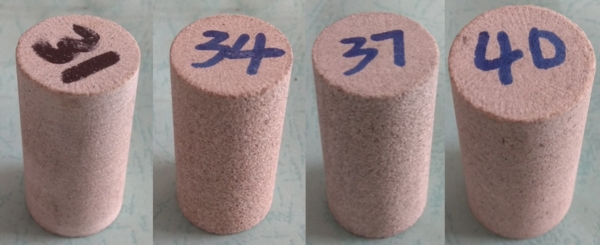
Tight sandstone samples.

The sample is taken from a gas field in the East China Sea, and the rock sample processing is carried out in strict accordance with the ISRM method recommended by international rock mechanics [40]. The average density of the sample is 2420 kg/m^3^. The sample size is 25 mm×50 mm cylinder. The surface of the rock sample is smooth without apparent defects, carefully grind both ends of the cylinder, and the non-parallelism and non-perpendicularity are controlled within ±0.2 mm to prevent the rock sample from affecting the test results due to factors such as uneven surface. The sandstone samples are shown in Fig. 2, the physical parameters are shown in Table 1.

**Table 1:** The physical parameters of the samples.

Experiment type	Confining pressure /MPa	Number	Height /mm	Diameter /mm
Uniaxial compression	/	31	50.2	25.2

Triaxial compression	20	34	49.96	24.94
	40	37	50.1	24.78
	60	40	49.9	24.98

### 2.3 Experimental scheme

This test carried out uniaxial and triaxial compression tests with confining pressures of 20 MPa, 40 MPa, and 60 MPa. Each experiment was conducted three times. For the triaxial compression test, the confining pressure is first loaded at the beginning of the test, and the target confining pressure is applied at a speed of 0.05 MPa/s by the hydraulic servo. The displacement control method loads the sample to failure at a 0.2 mm/min speed.

### 2.4 Experimental results

**Figure 3:**
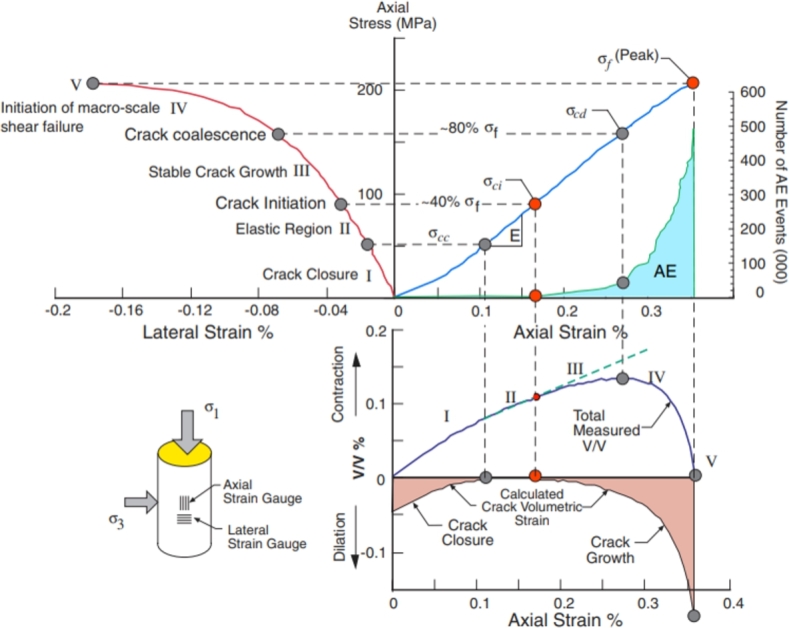
Stress–strain diagram of the rock showing the stages of crack development [41].

**Figure 4:**
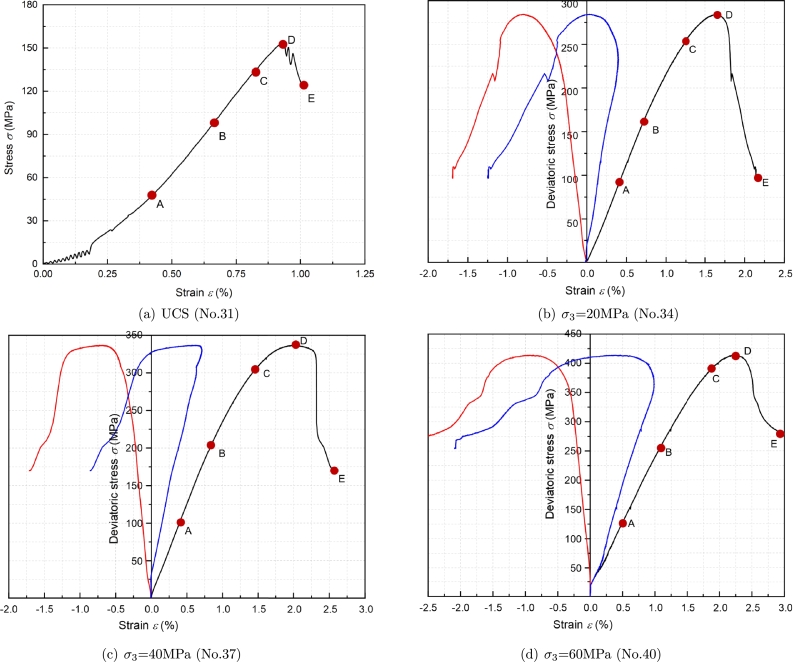
The stress-strain curve of the tight sandstone.

To study the process of the generation, propagation, and penetration of microcracks inside brittle rocks at different stress levels, Cai [41] divided the stress-strain curve of brittle rocks into five stages: Crack closure stage (Stage 1), Elastic stage (Stage 2), Stable crack growth stage (Stage 3), Initiation of macro-scale shear failure (Stage 4), Post-peak failure stage (Stage 5). In Cai's research, the development of rock microfracture was analyzed, so only the first four stages of the stress-strain curve were listed, as shown in Fig. 3. Stage 4 is also known as the Unstable crack growth stage [42]. The four stress characteristic points divide the curve into four stages. His research also provides the method for determining characteristic stress points. According to the methods of Cai et al., we divided the stress-strain curve of the tight sandstone obtained from the experiment into five stages, as shown in Fig. 4(a), 4(b), 4(c), 4(d).

The main characteristics of the five stages are as follows: In Stage 1: with the increase of confining pressure, the primary fractures inside the rock sample are compacted and closed, improving the integrity and stiffness of the rock sample. In Stage 2: Tight sandstone shows ideal linear elastic characteristics, and the increase of confining pressure makes the closure of original fractures more sufficient. The crack slip becomes more minor under load. Therefore, the axial deformation under low confining pressure is more significant than high confining pressure, and the elastic modulus increases with the confining pressure. In Stage 3: Randomly distributed microcracks gradually appear inside the sample, and the microcracks are independent of each other, with a relatively stable propagation rate. The propagation speed of cracks depends on stress conditions and the mechanical properties of the rock. Fig. 3 also shows a relatively small number of AE events during this stage. In Stage 4: The number of microcracks inside the sample increases significantly, and the microcracks connect, gradually forming macro fractures. The degree of damage and crack density of rocks may increase substantially at this stage. In Stage 5: The crack propagation speed reaches a critical state, leading to rock fracture and damage. The rock is destroyed due to its inability to withstand the load.

The sample has a noticeable confining pressure effect. It is mainly reflected in the increase of confining pressure, the peak strength and yield strength of samples increased significantly, and the elastic limit also increased significantly. Each sample produces a considerable radial strain after the peak. At higher confining pressure, the sample enters the residual stage after reaching the peak and particular residual strengths. The residual strength increases with the increase of confining pressure. The mechanical properties of tight sandstone are shown in Table. 2. The cohesion and friction angle of tight sandstone are 42.39 MPa, 37.73^∘^, respectively.

**Table 2:** Mechanical parameters of tight sandstone.

Confining pressure /MPa	Peak Strength /MPa	Elastic Modulus /GPa	Cohesion /MPa	Friction angle /^∘^
UCS	154.4	16.59	42.39	37.73
20	284.52	21.51		
40	336.83	23.21		
60	413.91	23.01		

The brittleness of a material is defined as the characteristic of crushing with almost no ductility or plastic deformation. The brittleness index can be used to evaluate the degree and effectiveness of fracture development in reservoir rocks, thereby helping to determine appropriate drilling and fracturing plans [43]. The brittleness index can also be used to evaluate the excavatability and fragmentation of rocks, helping engineers choose appropriate excavation methods and accurately evaluate the stability of rocks. It is equally important for geological hazard assessment and rock support design in certain engineering projects [44]. Therefore, accurately quantifying rock brittleness is of great significance for engineering practice. Currently, there are various methods for evaluating rock brittleness. Xu et al. [45] proposed a brittleness evaluation index Bp based on the pre-peak stress-strain curve and demonstrated its effectiveness through mechanical tests on different rocks. The definition of Bp is as follows [45] (Eq. (1)):

(1) $$B_{p}=M_{p}/E$$

**Figure 5:**
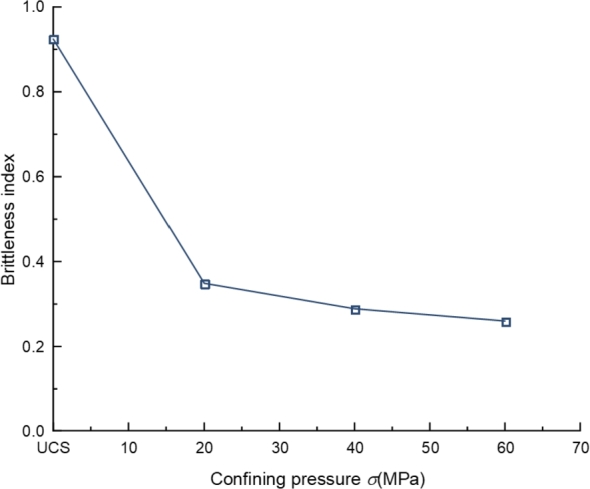
The relationship between brittleness index and confining pressure.

Where *E* and $M_{p}$ are the slopes of the curves for the Stage 2 (Elastic stage) and Stage 4 (Initiation of macro-scale shear failure) in Fig. 3. This study is based on the brittleness evaluation method proposed by Xu et al. and analyzes the sample's brittleness index. Fig. 5 shows the relationship between brittleness index and confining pressure. Previous scholars have adopted different methods for evaluating rock brittleness indices, and the obtained laws are consistent with those in this paper [43], [46].

Fig. 5 shows that confining pressure significantly impacts the brittleness index of rocks, and the brittleness index decreases with increasing confining pressure. Under lower confining pressure conditions, primary cracks or microcracks in rocks are prone to propagation, leading to fractures. When the confining pressure increases, the stress concentration phenomenon inside the rock will be alleviated, the expansion of cracks will be limited, and the deformation ability of the sample will be enhanced. Rock tends to undergo plastic deformation rather than fracture under stress, thereby reducing the brittleness index of the rock.

The brittleness of rocks under high confining pressure is relatively poor, unfavorable to the generation and expansion of hydraulic fracturing cracks. The higher the brittleness of the rock, the easier it is to form complex fractures during external interference or hydraulic fracturing. Therefore, in the fracturing construction of Petroleum engineering, attention should be paid to avoiding high-stress intervals, or corresponding technical measures should be taken in the fracturing construction, such as increasing the fracturing time, to improve the fracturing modification effect.

## 3 Numerical modeling and micro parameters calibration

### 3.1 Moment tensor algorithm

Cundall and Strack based on the DEM, the particle flow theory is established by introducing the idea of molecular dynamics [47]. The PFC can simulate the geometric heterogeneity of the medium according to the distribution of particle size, and study the fracture process of rock in detail. By establishing the particle flow model and defining the micro-parameters between particles, the changes of the basic properties of materials with the mechanical environment can be simulated. In addition, the bond between particles will fracture with the influence of load, so that the particles are separated from each other, to simulate the generation and propagation of cracks in rock. Therefore, it is very suitable for the study of rock AE mechanisms.

In seismology, the source information can be obtained by recording the source's released stress wave, and the source information can be studied by moment tensor theory [48]. Moment tensor is similar to stress tensor. Under the premise of certain failure criteria, different stress tensors can express different failure types. When using moment tensor to analyze the failure mechanism, it is necessary to decompose it according to the failure type and calculate the proportion of each failure type in the moment tensor of the event, so as to judge the failure type of the event.

In PFC, the moment tensor can be regarded as the corresponding displacement generated by all contact forces acting on the particle surface, which is equivalent to the same effect produced by physical force. The calculation process will be very complex if the recorded dynamic wave is converted into a moment tensor. The particle flow code can directly obtain the stress and motion of particles under load. Therefore, when new cracks appear, it is feasible to calculate the seismic moment by calculating the change of particle indirect contact force [49].

When the bond between particles breaks, the particles on both sides of the crack are bound to move, at the same time, the pores between particles in a specific area around the crack will deform, and the contact deformation will lead to the change of contact force. Therefore, the moment tensor can be solved by detecting the change of contact force and position of particles around the crack source. When calculating the moment tensor, it should be noted that an AE event may be composed of a single microfracture or multiple small-scale micro fractures in the actual rock failure process. When solving the moment tensor of the AE event composed of multiple bond fractures, the geometric center of the bond fracture should be regarded as the center of the AE event. The calculation formula of moment tensor component is as follows [36] (Eq. (2)):

(2) $${M_{i}}_{j}=\sum_{S}\left(\Delta F_{i}R_{j}\right)$$

The $\Delta F_{i}$ is the $i_{th}$ component of the contact force change, $R_{j}$ is the $j_{th}$ component of the distance between the contact point and the event centroid, the summation is performed in the range of the *S* surface.

The duration of AE is crucial to simulate the temporal and spatial distribution of AE events. In order to determine the duration of AE events, it is assumed that the source waves of all AE times in the simulation process are shear waves, the initiation and propagation velocity of microcrack is half of that of shear wave in rock. Then, the time required from the generation of microcracks to the propagation of shear waves to the boundary is $T_{s}$, then the duration of AE is $2T_{s}$. During each AE event, there are [50] (Eq. (3)):

(3) $$T_{s}=\frac{R_{a}}{V_{r}}$$

The $R_{a}$ is the average radius of source particles, $V_{r}$ is the shear wave velocity of rock at 2000 m/s in our study. During the failure of the rock, AE events are continuously generated, so the calculation of the moment tensor is also carried out in real time. The moment tensor varying with time can be obtained through this calculation method, but it will consume a lot of memory resources simultaneously. Therefore, the moment tensor with the largest scalar moment is selected to represent the moment tensor of this event. The scalar moment is calculated as follows [49] (Eq. (4)):

(4) $$M_{0}={\left(\frac{\sum_{j=1}^{3}M_{j}^{2}}{2}\right)}^{1/2}$$

The $M_{j}$ is the eigenvalue of the moment tensor matrix, then can calculate moment magnitude by the scalar moment.

In addition, the moment magnitude of AE events can be calculated by scalar moments. The moment magnitude reflects the failure intensity of the fracture event. The size of the dislocation of the fracture surface caused by each event and the released energy is characterized. The calculation formula is as follows [39] (Eq. (5)):

(5) $$M_{w}=\frac{2}{3}\log M_{0}-6$$

In order to physically distinguish which type of moment tensor corresponds to the source, some scholars proposed to decompose the moment tensor into three essential parts [51], [52]; Isotropic(ISO), Double couple(DC)and Compensated linear vector dipole (CLVD). DC and CLVD are called the partial part of the moment tensor. Feigner and young [53] adopted the ratio of isotropic part to biased part to quantify the failure type of the event (Eq. (6)):

(6) $$R=\frac{tr(M)\times 100}{\left|tr(M)\right|+\sum_{i=1}^{3}\left|M^{\prime }\right|}$$

**Figure 6:**
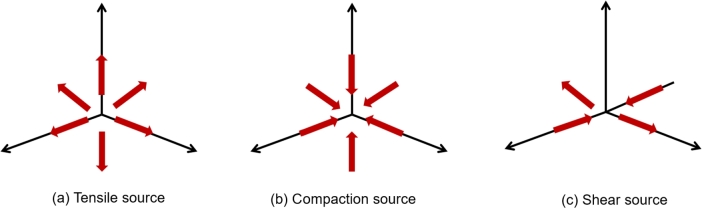
Diagram of AE sources type [54].

When the *R* >30%, it indicates that the source is mainly a tensile failure. When -30% < *R* < 30%, it is considered a shear failure. When *R* < -30%, it is a compaction failure. When *R* = 0, it represents a pure shear failure mechanism. The above methods have been widely used to explain the failure mechanism and predict the failure process of the rock mass. The failure source type of AE sources is shown in Fig. 6 (a, b, c).

### 3.2 Model building and calibration micro parameters

**Figure 7:**
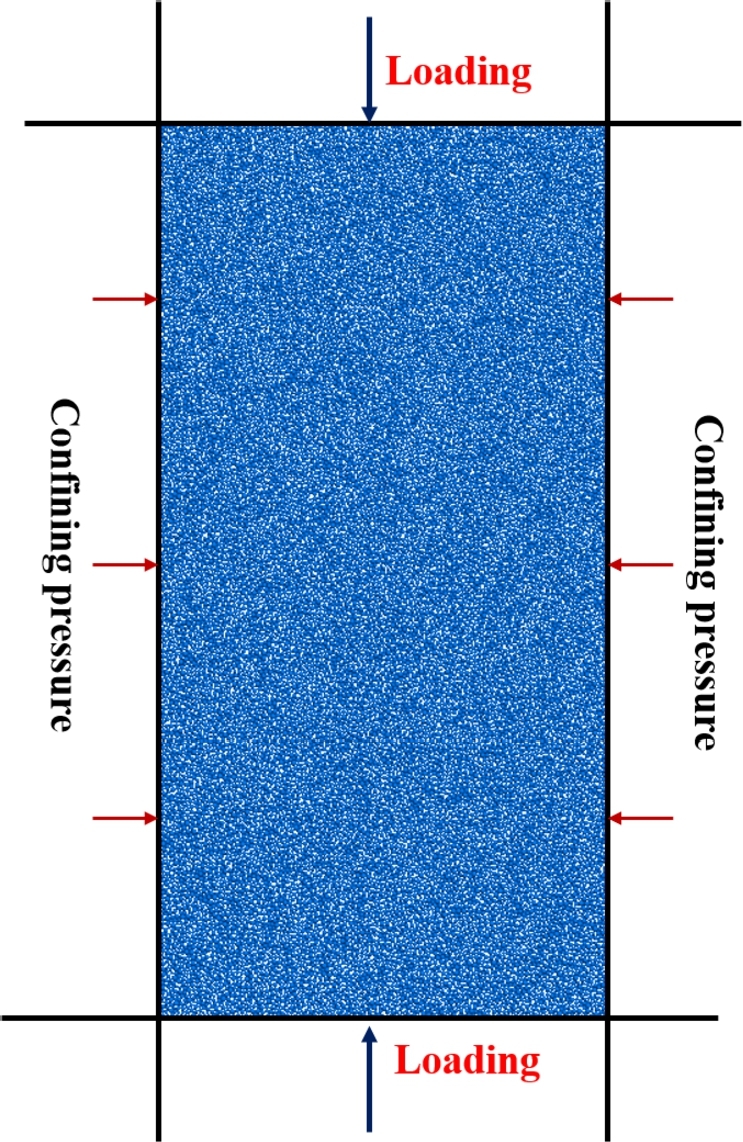
Numerical model of tight sandstone.

The size of the numerical model (The height and width are 50 mm and 25 mm, respectively, which is identical to the test sample) is selected according to the mechanical tests. The particle in the sample was generated using the radius expansion method. The same particle size and distribution were used for the specimen at different confining pressures. The particle size follows a uniform distribution, with $R_{min}$=0.2 mm and the particle size ratio is 1.66 [55]. This two-dimensional model comprises 20255 particles, the geometrical parameter as listed in Table. 3. The numerical model is shown in Fig. 7.

**Table 3:** Physical parameters of numerical model.

Model parameters	Value
Size (mm), *W*×*H*	25×50
Minimum particle size (mm), *R*_*min*_	0.2
Radius ratio, *R*_*max*_/*R*_*min*_	1.66
Porosity, *p*	0.16
Particle number	20255
Density, *ρ* (kg/m^3^),	2420

**Figure 8:**
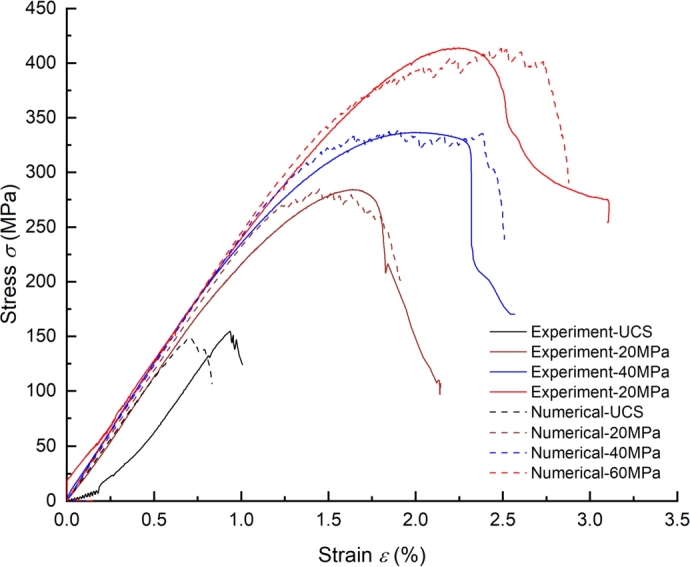
The stress-strain curve of experiment and numerical.

After the model is established, the confining pressure is applied to the model through the servo mechanism. When the confining pressure reaches the target value, the loading speed of 0.02 m/s for the upper and lower walls is given until the sample is damaged, the friction coefficient between the wall and the particle is 0. Obtaining a set of micro parameters that can accurately reflect the mechanical properties of materials is the key to simulation by DEM. According to the mechanical characteristics of tight sandstone, the contact bonds between the particles are designed as parallel bonds. The micro parameters are continuously adjusted by the trial and error method until the mechanical parameters obtained by numerical simulation agree with the experiment [56]. It should be noted that the linear parallel bond model in the DEM software regards the contact between particles as point contact, which overestimates the material's tensile strength. This problem can be effectively solved by modifying the moment contribution factor in the failure criterion of the parallel bond model [57], [58]. Fig. 8 summarize the stress-strain curve obtained by experiments and simulations. It can be seen that the numerical results are in good agreement with the experimental results. The determined microscopic parameters of tight sandstone are shown in Table. 4.

**Table 4:** Micro parameters of numerical model.

Parameter	Value
Effective modulus of the particle, *E*_*c*_ (GPa)	20
Stiffness ratio of the particle, *k*_*n*_/*k*_*s*_	2.5
Effective modulus of the parallel bond, *E*_*b*_ (GPa)	20
Stiffness ratio of the parallel bond, ${k_{n}}_{b}$/${k_{s}}_{b}$	2.5
Cohesion, *c*/MPa	135±30
Tension strength, *τ* (MPa)	135±30
Friction angle, *φ*/^∘^	40
Friction coefficient, *f*	0.5
Moment contribution factor, *M*	0

In the PFC, the simple spherical particle shape underestimates the constraint dependence of the peak strength, which leads to a high ratio of tensile strength to compressive strength [55], [59], [60]. Because the particles are regarded as rigid bodies, the numerical model cannot reproduce the compaction closure stage of natural rocks [61]. Differences in internal cracks between the numerical model and laboratory samples also contribute to the discrepancies [62]. These factors may cause errors between the sample and the numerical simulation results. Reference [49] provides methods to avoid these errors.

## 4 Numerical results

### 4.1 The spatial distribution of AE events

**Figure 9:**
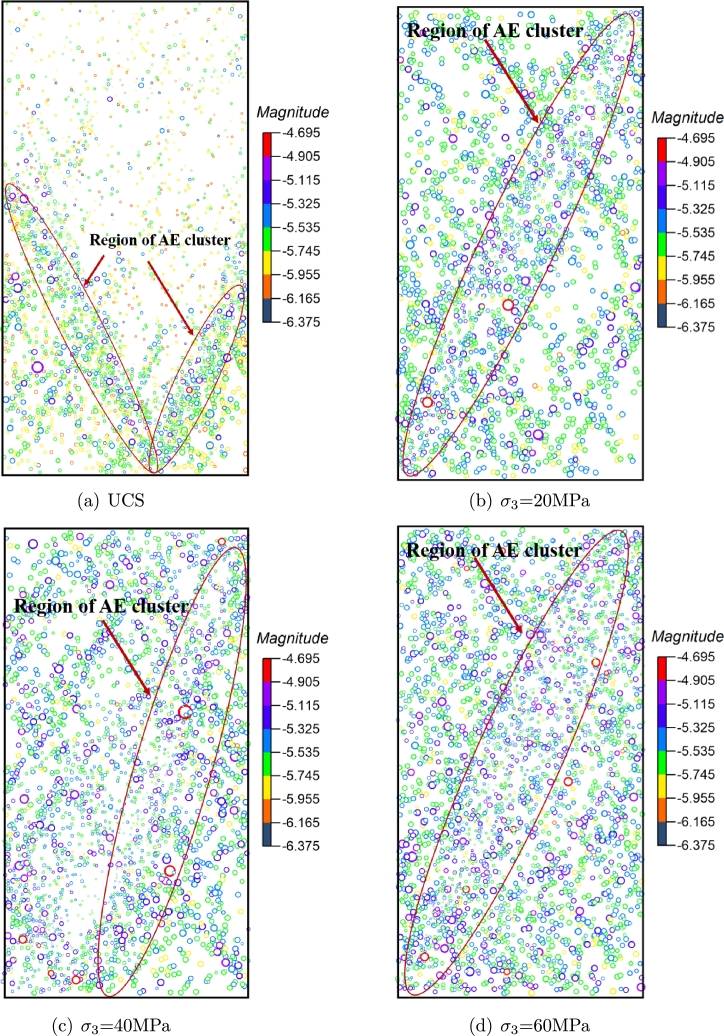
The distribution of AE events after loading.

The distribution of AE events after loading is shown in Fig. 9(a), 9(b), 9(c), 9(d). Circles with different radii represent the intensity of AE events. The larger the circle, the greater the influence range and intensity of the AE event. Different colors represent different moment magnitudes (i.e. rupture strength). The rupture strength of AE events under triaxial compression is higher than that under uniaxial compression. AE events are mainly concentrated on the shear plane of specimen failure. With the increase of confining pressure, the number of high-energy AE events in sandstone increases. In addition, the number of AE events around the shear surface also increases, and the spatial distribution of AE events effectively reflects the fracture information of tight sandstone. The influence of confining pressure on AE is very significant. With the increase of confining pressure, the AE characteristics of rock damage and deformation are very different from those under uniaxial compression.

Before the failure of the sample, a large number of cracks were produced in the model. The initiation, convergence and penetration of cracks resulted in the final failure of the sample. There are many small magnitude AE events on the macro shear surface. These small magnitude AE events are mainly caused by the shear slip of the macro fracture band during the failure process. Since these events occur after the failure of the specimen, their range and magnitude are small.

**Figure 10:**
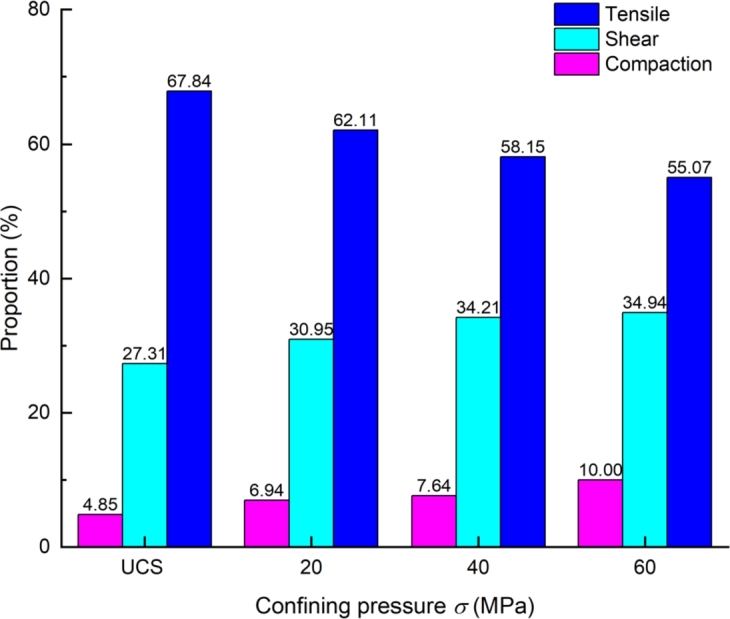
The proportion of AE source types.

The ratio of the AE failure sources at different confining pressure is shown in Fig. 10. The tensile failure is the main failure source, although the failure types of samples are splitting and shear. Kentaro et al. [63] measured by the sigma method, shear, tensile, and mixed events accounted for 62.78%, 16.6%, and 20.62%, respectively. In the Brazilian splitting test of Masayasu et al. [64], more than 50% of AE events are defined as tensile failure. It can be seen that there is no fixed proportion of failure types that produce AE during the loading to failure of the sample, and the proportion of each AE type is related to the loading conditions, that is, the mechanical environment of the rock mass.

For different confining pressures, the proportions of tensile failure were 67.84%, 62.11%, 58.15% and 55.07%, respectively. Due to the limitation of confining pressure, the proportion of compaction and shear source increases gradually. From 0 MPa (UCS) to 60 MPa, the proportion increases by about 7.63%, which is 1.28 times that under uniaxial compression. The proportion of shear failure increased from 27.31% to 34.94%, an increase of about 5.15%, indicating that shear failure is not the primary source of specimen failure.

### 4.2 The relationship between AE events and stress during loading process

**Figure 11:**
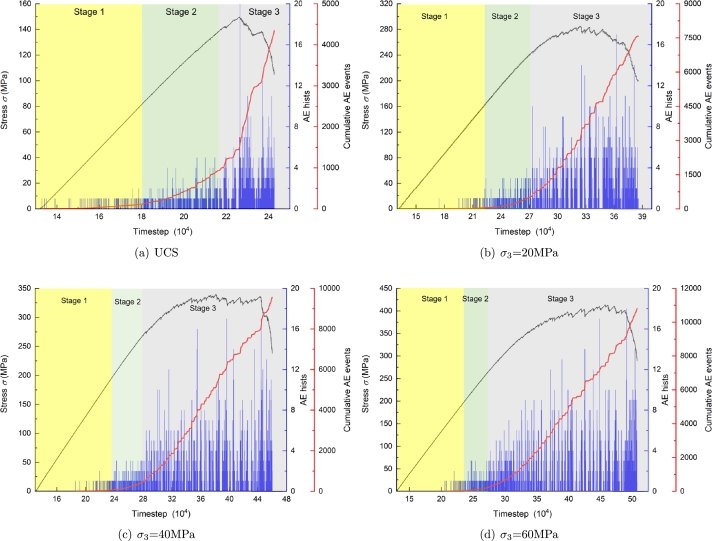
The relationship between AE events and stress with time.

The relationship between AE events and stress with time under uniaxial and triaxial compression is shown in Fig. 11(a), 11(b), 11(c), 11(d). According to the activity of AE events, the stress-strain curve can be divided into three stages: Quiet period (Stage 1): crack initiation stage; Active period (Stage 2): stable crack propagation stage; Burst stage (Stage 3): unstable crack propagation stage.

Under uniaxial and triaxial compression, the existence time of each stage is different. In the first stage, the stress-strain curve of the sample maintains a linear relationship. Due to the original pores, defects, friction between the end of the sample and the indenter, low-energy impact occurs from time to time in the crack initiation stage, for uniaxial compression, under the action of external load, the original defects and cracks in the sample can expand unrestricted, so there are more AE signals. The duration of the AE energy of triaxial compression is very short, the AE activity is weak, and it is quiet.

In the second stage, the cracks in the sample are further compacted under the action of external load, the AE concentration phenomenon is evident, many microcracks begin to nucleate and extent, and finally generate macro cracks, the interaction between the cracks is strengthened, but do not cause damage to the rock mass. In this process, the characteristic parameters of AE begin to change, and the number of AE events increases significantly. It should be noted that the number of large AE events (including multiple bond fractures) increases with the confining pressures.

With additional loading, the microcrack enters the stage of unstable propagation. The greater the confining pressure, the longer the duration of this stage. The stress decreases sharply with the deformation, the AE activity increases sharply compared with the first two stages, and the intensity also increases sharply. Macro fractures begin to appear in the rock, forming a shear fracture zone and leading to complete failure.

The distribution characteristics of AE time do not entirely correspond to the stress time characteristics. Near the peak value, the frequency of AE activity increases and the intensity increases significantly. However, AE burst's peak value lags behind stress's peak value. Before reaching the stress peak, there will be multiple microcrack accumulation areas in the sample, and the expansion trend of cracks becomes more intense. Due to the rock mass's heterogeneity and uneven stress distribution, stress concentration can quickly occur at the crack tip. Under stress redistribution, it will cause the fracture of adjacent units in this area. Many macro fractures have been formed in the rock sample, but the fractures are not connected. Therefore, although the AE time at this stage increases significantly, it does not reach the highest level.

After the peak load, the macro fractures begin to penetrate with the continuous application of external load. The area resisting microcrack propagation in the sample decreases, and the microcrack propagation shows an accelerated trend, the frequency and intensity of AE reach a peak. It is reflected from the side that if the AE technology is used to study the stability of mine rock mass and the emotional disaster caused by in-situ stress field, the failure time of rock mass is earlier than expected.

### 4.3 The spatial evolution of AE events at different stress levels

Studying the spatial distribution of AE under different stress levels is of great significance for predicting rock fracture instability. The above analysis shows that tight sandstone has different AE characteristics under uniaxial and triaxial compression. Therefore, this section will study the evolution law of AE events during sample failure to further reveal the failure characteristics of tight sandstone.

**Figure 12:**
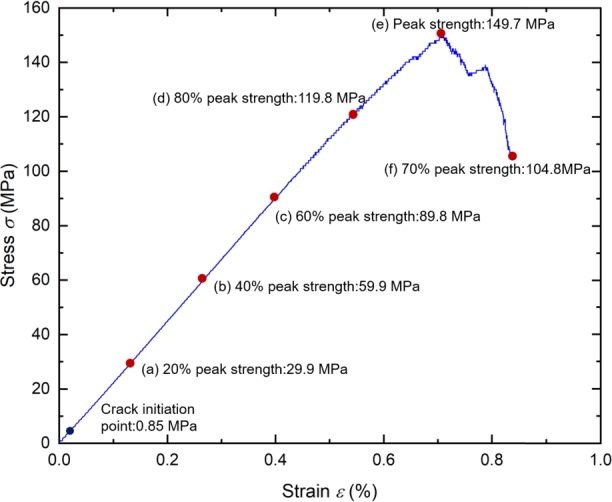
The stress-strain curve under uniaxial compression.

**Figure 13:**
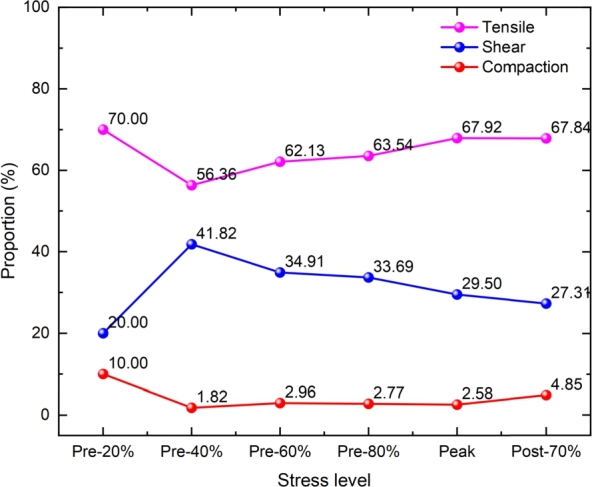
The proportion of AE failure source at different stress levels.

The stress-strain curve under uniaxial compression is shown in Fig. 12. The points in the curve represent different stress levels. The proportion of AE source types in total events under different stress levels is shown in Fig. 13. When the stress reached 20% of the peak stress, only ten AE events were generated in the sample, with the number of compaction, shear, and tensile failure sources being 1,2,7, respectively. Since the number of AE events is small, they are not statistically significant. Therefore, the proportion of changes in AE failure sources from 20% of the peak stress to 40% is not representative. As the load continues to be applied, overall, the AE failure sources shows the following trend from 40% of the peak stress to the end of loading: the proportion of tensile and compaction failure increases, and the shear failure decreases. From 40% of the peak stress to the end of loading, the shear failure decreased by about 14.51%, the compaction failure increased by 3.03%, and the tensile failure increased by 11.48% (The number of AE events at 20% of the peak stress is small, so there is no statistics.).

**Figure 14:**
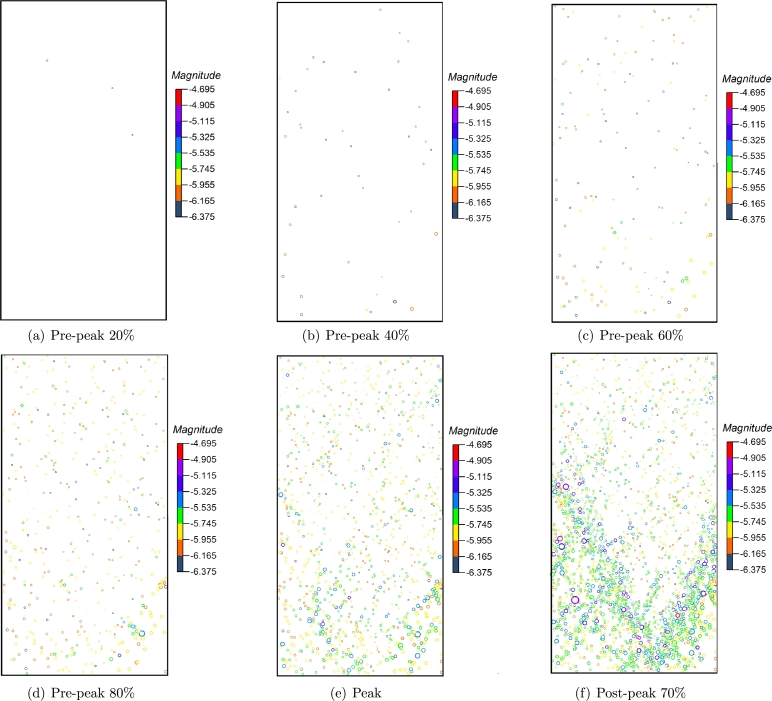
The distribution of AE evets at different stress level.

The spatial distribution of AE events under each stress level in Fig. 12 is shown in Fig. 14(a), 14(b), 14(c), 14(d), 14(e), 14(f). In the initial loading stage (within 20%), the AE events in the sample are few and scattered in the rock sample. The AE events in this stage are mainly caused by the rock sample's compression of original defects and microcracks. Most AE events comprise broken bond bonds, and only a few contain multiple microcracks.

When the stress reaches 40% of the peak stress, it is in the initial crack propagation stage, and an apparent increase of microcracks can be observed. When the stress reaches 60%∼ 80% of the peak stress, the AE events begin to concentrate in a small area, indicating that the stress concentration in this area is high and the AE events are caused by microcrack initiation and propagation is more. At this stage, the number of AE events increases obviously, and the radius of some circles begins to increase, indicating that some AE events magnitude begins to increase. AE events almost fill the sample when the peak stress is reached, and the crack expand from the initial failure position to the horizontal and vertical directions, respectively. There is still no central shear fracture zone in the sample. After the peak strength, the sample is destroyed, and the rock mass forms a macro discontinuity along the fracture zone. The initial crack in the rock mass has been penetrated, and no new AE event concentration area is generated. In the whole process, the rupture strength of AE is mainly concentrated in the range of -5.8 ∼ -5.2.

**Figure 15:**
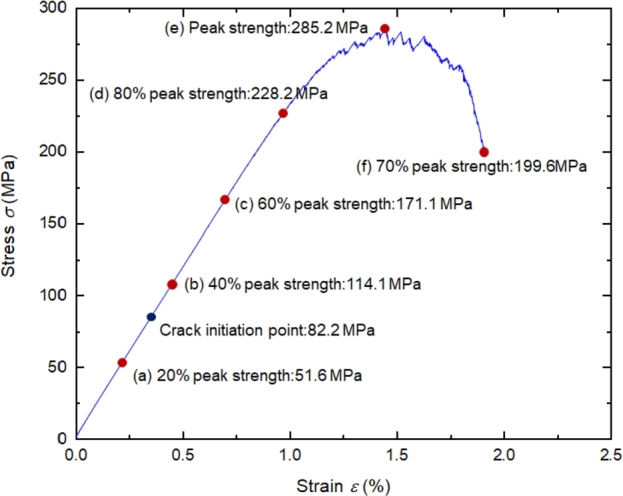
The stress-strain curve at 20 MPa.

**Figure 16:**
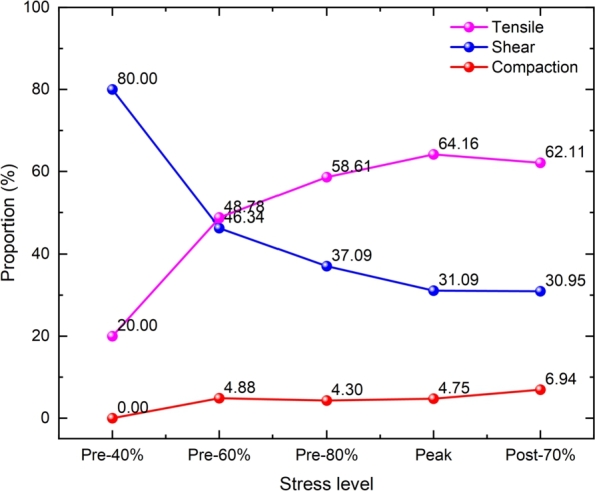
The proportion of AE failure source at different stress levels.

The stress-strain curve of the specimen under 20 MPa is shown in Fig. 15. The proportion of source of AE events in the total number under different stress levels is demonstrated in Fig. 16. AE events with and without confining pressure have different evolution laws. Compared with uniaxial compression, the variation range of each failure type under triaxial compression is more extensive. From the initial loading to the peak stage, the shear failure decreases with the stress level, and the tensile, compaction failures increase. The proportions of tensile, shear, and compression failure at the peak strength point are 64.16%, 31.09%, and 4.75%, respectively. After the peak strength, the proportion of compaction failure increases, the tensile and shear failure decreases slightly.

**Figure 17:**
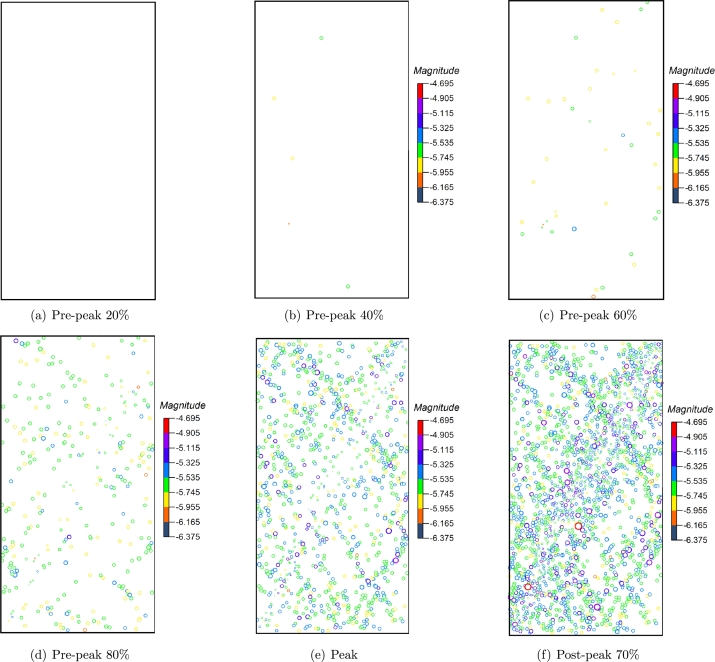
The distribution of AE types under different stress level.

Fig. 17(a), 17(b), 17(c), 17(d), 17(e), 17(f) shows the distribution of AE events at each point in Fig. 15, the intensity of the overall AE event is higher than that uniaxial compression, the moment magnitude is distributed between -5.6∼-5.0. Unlike uniaxial compression, a small number of AE events occur in the sample when the external load reaches 40% of the peak strength. The sources of AE events are mainly the closure and compaction of initial cracks, the failure of some rough surfaces after closure, and may also be caused by dislocation and slip between grains and a small number of friction activities. Under uniaxial compression, obvious AE will occur earlier due to the closure and compaction of microcracks at the initial stage of loading. In the triaxial compression test, AE at the initial loading stage will be restrained to a certain extent due to the damage healing during confining pressure loading. The greater the confining pressure applied, the more pronounced the inhibition. Therefore, in the initial stage of the triaxial compression test, less or no AE signal.

When the rock is loaded to 60% of the peak strength, the number of AE events of each type increases correspondingly but remains low. When the external load reaches 80% of the peak strength, the number of AE events increases significantly, especially the tensile failure, which increases by about 40% compared with the initial stage. The microcracks in the sample penetrate each other. Several prominent AE accumulation areas (mainly at the bottom and top of the sample) are formed in the rock sample. There are few AE events in the middle area of the sample, and there is a blank area of AE events, while the penetration of macro cracks occurs in the blank area, which is similar to the quiet period before a large earthquake. In this way, the location of rock crack propagation can be predicted. When the peak load is reached, obvious macro fractures are formed in the sample, the influence range and strength of AE are also enhanced. At post-peak 70%, the cracks at the bottom and top of the rock sample continue to expand to the middle. The cracks at the top and bottom penetrate, and an apparent macro fracture zone is formed in the sample, leading to the macro failure. A large number of small AE events have occurred around the macro fracture zone. These AE events are mainly formed after forming the macro fracture zone. Due to the continuous application of load and the shear friction of the macro fracture zone, these small AE events are usually difficult to capture in the experiment.

In the numerical test, the formation of a macro shear plane does not occur when the sample reaches the peak strength but occurs when the material deterioration accumulates to a certain extent with the continuous increase of load after the peak. At this time, the macro strain of the material belongs to the macro response of the micro-relief deformation of a single particle element.

Generally speaking, as a typical heterogeneous material, the mechanical properties of the internal unit particles of rock are quite different. In the loading process, the generation and propagation of microcracks in rock have certain randomness, so the spatial distribution of AE events also has randomness. Similarly, there are a large number of primary defects in the rock. Local stress concentration and microfracture areas will be generated when subjected to a small external load. However, the correlation between these micro fracture areas is small under a small stress field. The generation and expansion of a microfracture have no or little impact on the generation and development of other micro fractures. With increased stress levels, the interaction between microcracks far away increases. The propagation of a crack may lead to the propagation and generation of other cracks in a particular range. When the rock is close to instability and failure, the correlation of this stress field and the interaction range between cracks reach the maximum, and a minor external load disturbance will lead to the interconnection of many microcracks.

### 4.4 Effect of confining pressure on the frequency and magnitude of AE events

**Figure 18:**
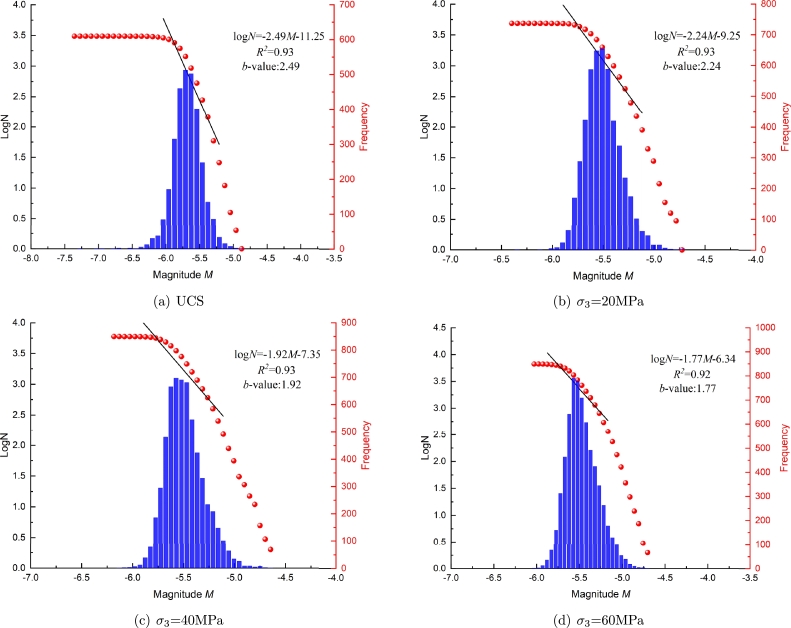
Relationship between the cumulative number of AE events, frequency and magnitude.

The relationship between AE events and magnitude is significant in analyzing rock AE characteristics. AE magnitude reflects the fracture strength of the event and the energy released by each crack. Fig. 18(a), 18(b), 18(c), 18(d) shows the relationship between frequency, magnitude and the cumulative number of AE events at different confining pressures. The relationship between AE frequency and magnitude obeys normal distribution. The maximum magnitude increases with the confining pressure. This indicates that the bearing capacity of rock is increasing. At the different confining pressures, the value of moment magnitude is mainly concentrated in the range of -5.75 ∼ -5.25. When the magnitude is greater or less than this range, the frequency of the magnitude is gradually decreasing.

As we all know, AE events are often caused by the convergence of multiple small-scale fractures inside the rock. A single micro-fracture may also produce an AE event. Therefore, a bonded broken may represent an AE event, and the rupture of multiple bonds may also constitute an AE event. However, these two scales of fractures have different AE magnitudes.

From Fig. 18, it can be observed that the cumulative number of AE events follows a power-law distribution with AE magnitude, which is consistent with the law obtained by previous researchers [39], [65]. Therefore, the moment magnitude and cumulative frequency also follow the power-law distribution. Gutenberg and Richter [66] put forward the famous G-R statistical relationship between earthquake magnitude and frequency (Eq. (7)):

(7) LogN=a−bM

Where *M* is the magnitude of AE events, *N* is the number of AE events with magnitude in △*M*, and *a*, *b* are constants. *b* value is an important parameter in AE research. Its physical significance is the ratio of small events to large events in the rock fracture process, reflecting the rock failure process. The change of *b* value is one of the crucial precursors of rock fracture.

To deeply analyze the failure characteristics of tight sandstone at different confining pressures, a straight line is fitted on the cumulative number of AE events, and the slope of the line is *b* value. The *b* value decreases with the confining pressures. From uniaxial compression to triaxial compression with confining pressure of 60 MPa, the value of *b* decreased from 2.49 to 1.74. This indicates that the AE events under uniaxial compression are mainly small, while those under triaxial compression are mainly large.

For the same axial stress, the larger confining pressure can make the effective shear stress of the fracture surface always negative [67]. The sliding of many fracture surfaces is limited. Due to the formation of a large number of microcracks in the sample, the micro-cracks cannot expand and penetrate in time under the inhibition of confining pressure, the AE activity and magnitude enhancement in the sample, resulting in a large number of large AE events in the model. Therefore, the *b* value is often small under high confining pressure.

**Figure 19:**
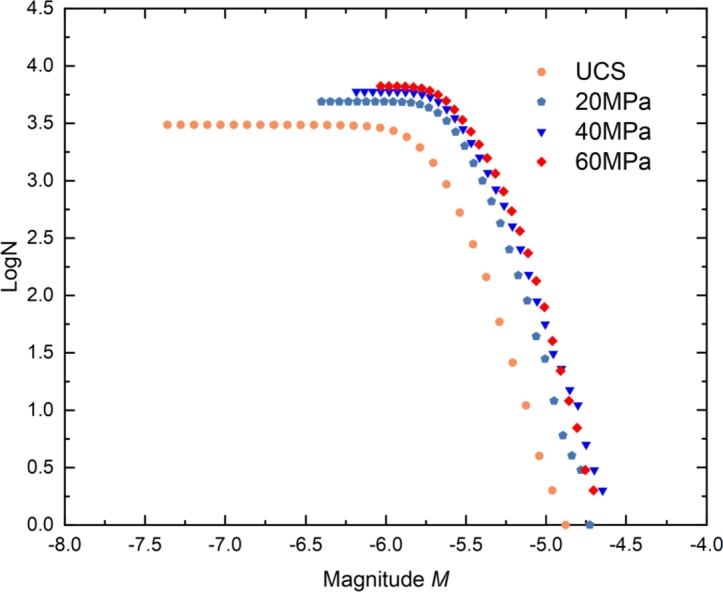
Relationship between the number of AE events and AE magnitude.

The relationship between moment magnitude and AE cumulative events is summarized in Fig. 19. The number of AE events and failure intensity increases with confining pressure. The relationship between the cumulative number of AE events and moment magnitude shows a logarithmic curve. For the uniaxial compression, the moment magnitude range is -7.4∼-4.8, and the average moment magnitude reaches the lowest level. This is because, under uniaxial compression, the failure probability of the specimen during loading is the highest.

When *M* < - 6.0, the number of AE events decreases with the moment magnitude, and the fitting slope of the cumulative AE event number curve is close to 0. In addition, when the confining pressure is 60 MPa, and *M* < - 5.25, the slope of the curve begins to decrease, which means that the moment magnitude and bearing capacity of AE events reach the maximum.

**Figure 20:**
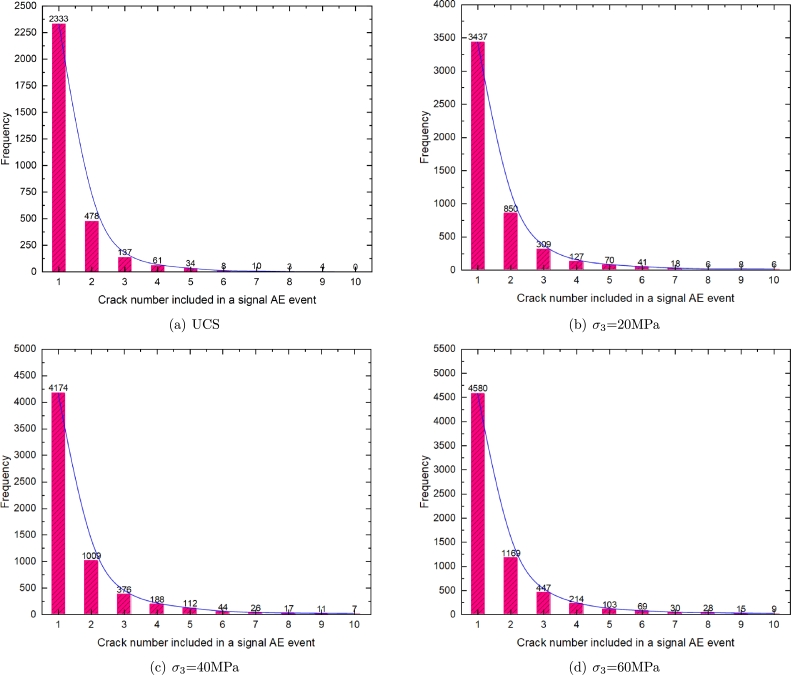
Relationship between the crack number included in a signal AE event and the AE frequency.

The relationship between the crack numbers of each AE event and the AE events is shown in Fig. 20(a), 20(b), 20(c), 20(d). It can be seen that the AE events decay exponentially with the number of microcracks. With the increase of the crack numbers of each AE event, the number of AE events decreases sharply. AE events are mainly concentrated in AE events with 1∼5 microcracks, ranging from 0 MPa∼60 MPa, accounting for 99.06%, 98.16%, 98.01% and 97.6% of the total events, respectively, it shows a decreasing trend with the increase of confining pressure. Under uniaxial compression, the number of AE events composed of a single microcrack is 2333, accounting for 75.94% of the total events. When the confining pressure is 60 MPa, the number of AE events consisting of a single microcrack is 4580, accounting for 68.64% of the total events. From 0∼60 MPa, the number of AE events in the range of 6∼10 is 25, 79, 105, and 151, respectively, accounting for 0.81%, 1.6%, 1.8%, 2.3% of the total number, respectively, showing an increasing trend with the confining pressures, with the increase of confining pressures, there are more and more large magnitude AE events.

## 5 Discussion

The traditional study of rock fracture AE characteristics only focuses on the analysis of the characteristics of the source AE signal and the location of the source without obtaining complete information on the type and direction of rock fracture. AE and moment tensor methods provide information from different angles, allowing for a comprehensive analysis of multiple aspects of rock damage. By combining these two methods, more comprehensive damage information can be obtained for evaluation from micro to macro scales to understand better the interaction between microcracks in rocks and their propagation and connection mechanisms. Zou et al. [68] evaluated the effectiveness of deep hole blasting technology in controlling strong earthquakes using AE and moment tensor methods. Zhou et al. [69] analyzed the development of seepage channels in underground mining engineering using moment tensor inversion and acoustic emission monitoring. Wu et al. [70] studied the fracture propagation mechanism in shale oil reservoir. Ren et al. [71] evaluated the spatiotemporal evolution and damage of microcracks in schists under true triaxial compression using AE localization technology and moment tensor analysis. The above studies indicate that the reasonable use of AE and moment tensor inversion methods can provide important support and guidance for a deeper understanding of rock damage mechanisms and engineering practices. The moment tensor inversion results also confirm that the decreasing isotropic component and the increment of double-couple can be used as the precursors of rock failure [25].

Researchers can obtain information on the stress state of internal rock fractures by combining AE testing with moment tensor inversion. This is helpful in understanding the rock fracture mechanism, including fracture mode, stress concentration area, and fracture propagation path. By monitoring and analyzing the AE activities during rock failure and identifying key fracture events and failure mechanisms, important clues can be provided for the safety assessment of engineering structures. In addition, by studying the spatial distribution and characteristics of AE events, engineers can understand the fracture evolution of rocks, predict possible instability areas, and design construction schemes accordingly.

However, there are still some controversies and limitations regarding the use of AE and moment tensor methods in determining the nature and location of rock damage, such as: (1) For complex rock systems and loading conditions, there may be some uncertainty in directly linking AE and moment tensors to specific damage properties. This is because the damage behavior of rocks is influenced by various factors, such as the heterogeneity of the rock itself, pore structure, and stress distribution, which may lead to differences between AE results and actual damage properties [72]. (2) AE localization is determining the location of damage sources by recording the arrival time of AE events. Due to the heterogeneity of rocks and the complex propagation path of sound waves, the accuracy of AE localization poses certain challenges [27].

In addition to the above factors, indoor AE testing has the following limitations: (1) More than 90% of AE tensile failure sources cannot be captured [39], [49]. (2) Incomplete recording of AE signal at peak stress [31]. The AE simulation method based on moment tensor inversion can simultaneously provide the characteristics of the time, space, and fracture strength of AE events, reproduce the process of rock crack initiation, development, and penetration, reveal the failure mechanism of rocks, and is an excellent supplement to indoor AE experiments. The research results of Zhang et al. [73] indicate that the numerical simulation results of AE based on moment tensor inversion are in good agreement with indoor experimental results. The research results can provide a basis for studying rock failure mechanisms and identifying and verifying AE characteristics in the laboratory or on-site. Zhang et al. [74] monitored the initiation and propagation process of hydraulic fractures based on AE technology and identified weak surface failure properties based on moment tensor inversion. Two possible modes of interaction between hydraulic fractures and weak planes were observed. The research results have important references for fracturing construction design.

The AE research results based on moment tensor inversion can provide important data support for improving rock mechanics theory. Through comparison and verification with the actual AE data, researchers can optimize and enhance the existing mechanical model to more accurately describe the rock failure behavior and mechanical response and develop a more accurate AE positioning algorithm, thus providing new ideas and methods for rock mechanics theory and geological disaster prediction.

It should be noted that this study adopts a method based on moment tensor inversion to study the AE characteristics of rocks. Khazaei et al. [75] proposed a new approach to calculating AE magnitude based on strain energy in PFC. This method can obtain more real AE events, but the magnitude obtained is still larger than the actual [65]. The reasons for the unreasonable magnitude can be attributed to the following [75]: (1) In actual AE testing, irregular rupture at the source can affect the propagation of sound waves and lead to the occurrence of small magnitude events. (2) Due to indoor acoustic emission testing limitations, not all acoustic emission events are recorded, while numerical simulations record all events. (3) The fracture of inter particle bonding and the decrease in stress in numerical simulation are instantaneous, resulting in excessive energy release. (4) Spherical particles cannot reflect the heterogeneity of actual mineral particles. Therefore, there is still a lot of work to be done to accurately and reasonably simulate the AE magnitude.

## 6 Conclusion

Considering some limitations of indoor AE experiments, such as the sensitivity of the AE probe and it is easy falling off during the loading, a numerical model of tight sandstone is established to analyze further the failure mechanism of tight sandstone. Numerical simulation is carried out to deeply investigate the AE characteristics of tight sandstone under uniaxial compression and different confining pressures. The following conclusions are obtained:

(1) The average elastic modulus of the sample is 21.08 GPa, the cohesion is 42.39 MPa, and the friction angle is 37.73^∘^. The brittleness index of the sample decreases with the increase of confining pressure. In rock failure, AE activities show different characteristics with increased stress. The occurrence of AE events is mainly caused by crack propagation. In the initial compaction and elastic deformation stage, AE activities are not obvious, and moment magnitude is low. At the stage of stable propagation, the cracks change from stable to rapid growth, and the AE activity becomes significant. In the microcrack agglomeration and explosion stage, the AE activity is very active, which leads to the final instability and failure of the specimen.
(2) In the whole failure process, AE events are mainly tensile failures, although the main failure types of samples are splitting and shear failure. The failure types of AE events at different stress levels and sample failure have no fixed proportion. They are not related to the stress level but the mechanical environment of rock materials.
(3) The spatial distribution of AE intuitively reflects the evolution law of crack initiation, propagation, and penetration in the progressive failure of rock. Microcracks first occur at the top and bottom of the sample and expand inward along the loading direction. Empty blank areas of AE events can quickly appear in the sample's middle. The propagation direction of cracks can be judged with the help of the blank spaces of AE events, then the failure mode of the rock sample is predicted.
(4) The relationship between the AE frequency and magnitude presents a normal distribution, and the cumulative number of AE events and magnitude presents a power-law distribution. The *b* value decreases with the increase of confining pressure. The AE events at high confining pressure are mainly high magnitude events.

## CRediT authorship contribution statement

Yike Dang: Performed the experiments. Analyzed and interpreted the data. Wrote the paper. Zheng Yang: Contributed reagents, materials, analysis tools or data. Wrote the paper. Haiyan Zhu: Conceived and designed the experiments.

## Declaration of Competing Interest

The authors declare that they have no known competing financial interests or personal relationships that could have appeared to influence the work reported in this paper.

## Data Availability

Data will be made available on request.
